# Short-term exposure with vibration and its effect on attention

**DOI:** 10.1186/s40201-014-0135-1

**Published:** 2014-11-13

**Authors:** Zahra Zamanian, Asghar Nikravesh, Mohammad Reza Monazzam, Jafar Hassanzadeh, Mohammad Fararouei

**Affiliations:** Department of Occupational Health, School of Health, Shiraz University of Medical Sciences, Shiraz, Iran; Department of Ergonomics, School of Health, Shiraz University of Medical Sciences, Shiraz, Iran; Department of Occupational Health, School of Public Health, and Center for Air Pollution Research (CAPR), Institute for Environmental Research (IER), Tehran University of Medical Sciences, Tehran, Iran; Department of Epidemiology, School of Health, Shiraz University of Medical Sciences, Shiraz, Iran

**Keywords:** Accidents, Whole body vibration, Neuropsychological tests, Attention, Concentration

## Abstract

**Background:**

About 20 to 50 percent of accidents are due to some forms of carelessness and mindedness. Studies suggested that vibration is one of the most important physical factors in human performance when traveling on any sort of vehicles. The aim of this study was to investigate the effect of vehicles vibration on cognitive performance (attention and concentration).

**Methods:**

The sample consists of 25 male students who undergone 4 experimental phases: acceleration low vibration (0.53 m/s^2^), medium vibration (0.81 m/s^2^), high vibration (1.12 m/s^2^) and non- exposure to vibration (control phase) according to ISO 2631–1 standard and with fixed frequency between 3 to 7 Hz. Students paired T test was applied to analysis the data, using SPSS software ver. 19.

**Results:**

The mean number of correct answers obtained from selective attention test under vibration levels of 0.53 m/s^2^ and 0.81 m/s^2^ were significantly lower than the condition without vibration. The mean reaction time of divided attention test in three vibration levels was significantly lower than the condition without vibration. Exposing to vibration reduces number of correct answers, selective attention and reaction time.

**Conclusion:**

In this study, it was found that vibration may disrupt the ability of the participants for sustainability attention. It also changes the precision and speed of humans’ information processing systems.

## Background

Driving accidents are considered as one of the main challenges in our developed world, especially in Iran [1]. According to the World Health Organization reports in 2013, more than one million and two hundred thousand people are killed in road accident and more than fifty million people have subjected to serious damages annually [2]. Also other reports have suggested that road accidents will become to third causes of death and disability in human societies by 2020 [3]. Based on the reports of Statistics Maintenance and Transport Organization of Iran, The death toll road are 26,089 persons and stats of wounded was increased to more than 245,000 people in 2004 [4].

Many researchers have pointed out that human factors are the causes of more than 70 – 75 percent of road accidents [5,6]. Vehicles and road factors both have a same contribution percentage in road accidents (%10-15) [7,8]. Among human factors, attention and concentration are considered as the most important cognitive components in road accidents, so that 20–50 percent of incidents include some forms of inattention and distraction [9]. Vibration is one of the most important physical factors in all vehicles [10-12]. Harisson [13] and Kuiper [14] showed that the vibration frequency of most vehicles ranges between 3 to 7 Hz. Previous studies have shown that attention is a kind of mental and physical efforts for environmental stimuli and it is necessary for conscious perception and quick responses to various environmental stimuli and making adjustments to environmental condition. Attention contains all information relating to memory, perception, and other cognitive processes. Attention allows people to rationally apply their limited cognitive resources and memorize important information [15]. One of the major roles of attention is recognizing objects and events in environment [16,17].

Subjects and important event’ detection are one of the most important applications of attention. Therefore, attention can be divided into two types: selective attention (centralized) and divided attention (sporadic). The evaluation of these two types specially based on different functions is very critical. If it is not considered seriously, it can lead to injury or high losses [18].

Several researches have studied the effects of vibration on reaction time, workload, information processing and visual performance but no research has been studied the effects of vibration on cognitive performance specially on attention and concentration. This study is designed to assessment the effects of vibration on attention using neuropsychological tests.

## Methods

This study is a kind of semi-experimental interventional, before - after study that was designed to investigate whole body vibration’s influence on human cognitive function.

### Participants

In this study, twenty five 20–30 years old male students having sight 20/20 and other required criteria have been selected. Test site was physical-agents laboratory of school of public health in Tehran University of Medical Sciences. It should be noted that this study was conducted in accordance with the Helsinki Declaration of 1964 as revised in 1984. Additionally, all participants signed an informed consent form before commencement of the study.

### Experimental setup

Since the aim of this study was to simulate and evaluate the effects of whole body vibration on human cognitive function as it would happen on a truck, the acceleration and frequency of vibration were set similar to trucks. In the present research, all experiments were conducted using a vibration simulator which produces vibration as sinusoidal/random waves in three directions (X, Y and Z axis) and different acceleration rates and frequencies.

Drivers were connected to vehicle’s chair using a metal base. The vibration rate was regulated via LABVIEW software in a range of frequency from 3 to7 Hz and acceleration rates of 0.53, 0.81, and 1.12 m/s^2^. A whole body vibration meter was used to calibrate the vibration simulator machine.

‘Human Response Vibration meter’ (Brüel & Kjær 2512) was used to measure the vibration.

### Experimental procedure

Cognitive tests including selective attention and divided attention tests were performed before exposure to vibration. This type of intervention was so that participants were exposed to vibration in three different accelerations. In this research, randomized selection was performed to remove the sequences effects so that the 1.12, 0.81, 0.53 m/s^2^ accelerations were considered for each participant. Some tests started with acceleration of 0.53 m/s^2^, while another ones with accelerations of 1.12, or 0.81 m/s^2^.

In the next stage, each person was exposed to different acceleration for 1 to 2 minutes, and then in the third minutes cognitive tests were performed again. Then the vibration simulator was regulated in acceleration kind 2. The method was repeated for other accelerations. It should be noted that a five minutes rest was considered between different tests for eliminating of previous vibration exposure. Then cognitive testing results which obtained before and after vibration exposures were compared to each other and finally error rate was computed.

In this study “selective attention test” was used to assessment selective attention. On the computer screen, different alphabet letters were displayed continuously and the participant was asked to carefully look at them and then push ‘space bar’ key only if see “S” or “M”. The result of selective attention test was reported according to the number and reaction time of selective attention in millisecond for each subject. Gualtieri and Johnson [19] have reported the reliability and the validity of these tests in the range between %81 -%85.

In present study, the divided attention in participants was studied by “divided attention test” in which the alphabet letters displayed continuously in a random, and participant should be pushed “?” button If sees “M” letter on The right of the screen. Also if the participant sees “S” letter on left of the screen, he should push “Z” button. The result of divided attention test was reported according to the number and reaction time of divided attention in millisecond. Reliability and the validity of these tests were determined in the range between %81 -%85 [19].

### Statistical analysis

Students paired T test was applied to analysis the data, using SPSS software ver. 19.

## Results

Table 1 presents the mean and standard deviation of reaction time and correct answer obtained from selective attention test under 0.53, 0.81 m/s^2^ and 1.12 vibration accelerations and 3–7 Hz frequencies. As seen from Table 1, the mean number of correct answers under vibration levels of 0.53 m/s^2^ and 0.81 m/s^2^ were significantly lower than the condition without vibration (P_value_ = 0.001). Under vibration conditions 1.12 m/s^2^, the relationship between the number of correct answers with vibration was not significant (P_value_ =0.186).

**Table 1:** **Results of selective attention test**

**Variables**	**Acceleration (m/s** ^**2**^ **)**	**Exposed group**	**Control group**	**P-value**
		**Mean (±SD)**	**Mean (±SD)**	
**Reaction time (ms)**	0.53	431.35 ± 29.29	432.09 ± 37.40	0.885
**Correct answers**		53.95 ± 2.06	53.56 ± 3.45	0.001
**Reaction time (ms)**	0.81	428.35 ± 29.29	432.09 ± 37.40	0.662
**Correct answers**		52.46 ± 2.06	53.56 ± 3.45	0.001
**Reaction time (ms)**	1.12	429.85 ± 29.29	432.09 ± 37.40	0.662
**Correct answers**		53.95 ± 2.06	53.56 ± 3.45	0.186

The results of repeated measures analysis revealed that the effect of vibration (in three accelerations) on reaction time of selective attention was not significant (P_value_ = 0.517), but the effect of vibration on number of correct answers was significant (P_value_ = 0.001). In other words, participants who were exposed to vibration had lower correct answers.

Table 2 provides the results of divided selective attention test. The mean reaction time of divided attention test in three vibration levels 0.53 m/s^2^, 0.81 m/s^2^ and 1.12 m/s^2^ was significantly lower than the condition without vibration (P_value_ =0.000, P_value_ =0.005, P_value_ =0.001). Also the mean number of correct answers in vibration levels of 0.53 m/s^2^ (P_value_ =0.001), 0.81 m/s^2^ (P_value_ =0.001) and 1.12 m/s^2^ (P_value_ =0.003) was significantly higher than the condition without vibration.

**Table 2:** **Results of divided attention test**

**Variables**	**Acceleration (m/s** ^**2**^ **)**	**Exposed group**	**Control group**	**P-value**
		**Mean (±SD)**	**Mean (±SD)**	
**Reaction time (ms)**	0.53	596.94 ± 76.42	617.01 ± 84.89	0.005
**Correct answers**		49.1667 ± 6.04	44.12 ± 7.18	0.001
**Reaction time (ms)**	0.81	593.94 ± 76.42	617.01 ± 84.89	0.001
**Correct answers**		46.17 ± 6.04	44.12 ± 7.18	0.001
**Reaction time (ms)**	1.12	595.44 ± 76.42	617.01 ± 84.89	0.001
**Correct answers**		47.67 ± 6.04	44.12 ± 7.18	0.003

The results of repeated measures analysis shows that the effect of vibration (in three acceleration) on reaction time and correct answers of divided attention was significant (P_value_ = 0.003 and 0.001 respectively); it means that subjects with vibration exposed had lower reaction times and correct answers than the others (no vibration exposed).

## Discussion

In the present study, participants were subjected to four stages of selective attention and divided attention tests according to 3 vibrations levels: low (0.53 m/s^2^), Average (0.81 m/s^2^), High (1.12 m/s^2^) and without exposure to vibration (control status) based on Classification Standard ISO2631-1 and 3–7 Hz fixed-frequency. Considering the results of selective attention and divided attention tests participants in control and intervention groups, it can be stated that vibration disrupts the sustainability of individuals. Also vibration increases the speed and precision of information processing system. This result is consistent with the findings of Miyamoto et al. [20]. They have showed that vibration have significant effect on the mental workload. Mental workload reaches its peak value in high vibrations. This is consistent with the mentioned studies [20]. The results of Sundstrorm et al. in Sweden showed that the maximum disorder rate in reading and writing performance ranges between 2–4 Hz [21]. Results of Bhiwapukar et al. [22] in Japan showed that the maximum interference rate in visual performance ranges between 2–12 Hz. Vibration in X direction had more influence on visual performance. The highest effect on reading performance was reported in 4 Hz frequency. The results of Gualtieri and Johnson also suggested that body vibration in 2.5 Hz frequency causes disorder in the visual performance of participants [19].

This is in contrast to Newell’s results. Based on the results of Newell et al. [23], exposure to vibration and awkward posture makes some disorders in human concentration, and consequently increases the reaction time. In this study, tests were performed based on Iso13090-1 standard. First, the participants were under in no vibration statue and then for 3 minutes be under vibration with 1–20 frequency Hz and with 1.4 m/s^2^ intensity average and 1.1 m/s^2^ in Z axis and in last minutes doing the visual reaction time test [23].

Manser et al. [24] stated that vibration hasn’t any effect on reaction time, the attempt rate of participants and number of correct answers. They applied vibration of train in 3 levels and 3 directions X, Y, Z. Participant performed a working memory test [24]. It is worth to note that the different frequencies, accelerations, and tests were used in researches mentioned above. Miyamoto et al. [20] found that increased mental workload can reduce the level of cognitive activity and task.

The results of the present study are consistent with Lundstro et al. [25]. They have revealed that whole body vibration with low frequency can lead to increased drowsiness and loss of consciousness. They have examined 30 people using whole body vibration in 2–20 Hz frequency and 0.3 m/s^2^ acceleration. Their electroencephalography and electrocardiogram results showed that vibration can increase the activity level of beta brainwaves, and reduce alpha waves activity and heart rate [25].

One of the limitations of the present study was that it was conducted on the participants within a specific age range in controlled conditions, while vibration in real fields can have different effects depending on various individual and environmental factors.

Yet, the strong point of the study is that it is the first research investigating the effect of vibration on divided and non-divided concentration in Iran.

## Conclusions

Based on our findings, vibration can disturb the stability of attention and increase the drowsiness and mental load; and these issues are so critical in driving, since 20 to 50% of car accidents involved lack of attention stability and distractibility [7]. In this study, the neuropsychological standard test was used which is a valid test in the psychiatry and psychology.

In regards to improving the speed and precision of information processing systems, it can be pointed out that a high level of vigilance is required for doing the divided attention test, so participants carefully performed their test.
